# Induction of omega 6 inflammatory pathway by sodium metabisulfite in rat liver and its attenuation by ghrelin

**DOI:** 10.1186/s12944-015-0008-3

**Published:** 2015-02-17

**Authors:** Sevim Ercan, Ceren Kencebay, Goksun Basaranlar, Filiz Ozcan, Narin Derin, Mutay Aslan

**Affiliations:** Akdeniz University, Vocational School of Health Services, Antalya, 07070 Turkey; Akdeniz University, Medical School, Department of Biophysics, Antalya, 07070 Turkey; Akdeniz University, Medical School, Department of Medical Biochemistry, Antalya, 07070 Turkey

**Keywords:** Sodium metabisulfite, Ghrelin, Polyunsaturated fatty acids

## Abstract

**Background:**

Sodium metabisulfite is commonly used as preservative in foods but can oxidize to sulfite radicals initiating molecular oxidation. Ghrelin is a peptide hormone primarily produced in the stomach and has anti-inflammatory effects in many organs. This study aimed to assess endogenous omega-3 (n-3) and omega-6 (n-6) polyunsaturated fatty acids (PUFAs) in rat peripheral organs following sodium metabisulfite treatment and determine the possible effect of ghrelin on changes in n-6 inflammatory pathway.

**Methods:**

Male Wistar rats included in the study were allowed free access to standard rat chow. Sodium metabisulfite was given by gastric gavage and ghrelin was administered intraperitoneally for 5 weeks. Levels of arachidonic acid (AA, C20:4n-6), dihomo-gamma-linolenic acid (DGLA, C20:3n-6), eicosapentaenoic acid (EPA, C20:5n-3) and docosahexaenoic acid (DHA, C22:6n-3) in liver, heart and kidney tissues were determined by an optimized multiple reaction monitoring (MRM) method using ultra fast-liquid chromatography (UFLC) coupled with tandem mass spectrometry (MS/MS). Cyclooxygenase (COX) and prostaglandin E2 (PGE2) were measured in tissue samples to evaluate changes in n-6 inflammatory pathway.

**Results:**

Omega-6 PUFA levels, AA/DHA and AA/EPA ratio were significantly increased in liver tissue following sodium metabisulfite treatment compared to controls. No significant change was observed in heart and kidney PUFA levels. Tissue activity of COX and PGE2 levels were also significantly increased in liver tissue of sodium metabisulfite treated rats compared to controls. Ghrelin treatment decreased n-6 PUFA levels and reduced COX and PGE2 levels in liver tissue of sodium metabisulfite treated rats.

**Conclusion:**

Current results suggest that ghrelin exerts anti-inflammatory action through modulation of n-6 PUFA levels in hepatic tissue.

## Introduction

Sodium metabisulfite (Na_2_S_2_O_5_) is one of the leading food preservatives and is used for the preservation of pastries, cheese, beverages, ground beef, margarine, fruit, sausages, sweets and fish [1]. It serves to prevent growth of bacteria, mould, yeast and controls enzymatic and non-enzymatic browning [2]. When ingested, Na_2_S_2_O_5_ reacts with water leading to the generation of bisulfite (HSO_3_^−^), sulfur dioxide (SO_2_) and sulfite (SO_3_^2−^) [3]. Thus, Na_2_S_2_O_5_ is termed as a “sulfating agent” because it releases SO_2_.

Ingested Na_2_S_2_O_5_ is absorbed in the gastrointestinal tract and is distributed to all tissues via systemic circulation [4]. Many organs are protected against the harmful effects of sulfite by the detoxifying sulfite oxidase, which oxidizes sulfite to sulfate [5]. Exogenous sulfites are presented to the liver’s biotransformation system for processing and elimination and their oxidation is diffusion limited [6]. However, when in excess amount they can stress the detoxification capability of the liver or be partially processed and accumulate in the liver and adipose tissue [2]. This can lead to increased liver stores of these toxic compounds and cause tissue injury. Studies have shown that sulfite oxidation can cause oxidative damage in organs such as liver and kidney [7].

Previous studies have shown that long-term in vivo exposure to sulfite aerosols induces inflammatory reactions [8,9] and that alveolar macrophages incubated with sulfite generate significantly increased amounts of arachidonic acid (AA) and AA-derived eicosanoids synthesized by cyclooxygenase (COX), such as prostaglandin E_2_ (PGE_2_) and platelet aggregating thromboxane B_2_ (TXB_2_) [10]. Indeed, different response patterns induced by sulfur-related compounds may be due to changes in the generation and release of inflammatory mediators which play an important role in eliciting reactions in tissues and cells. In vitro studies provide evidence that sulfite is able to activate alveolar macrophages by lipid mediators such as platelet-activating factor (PAF) and leukotriene B4 (LTB4) [11,12].

Ghrelin is an acylated peptide that stimulates the release of growth hormone (GH) from the anterior pituitary via binding to the GH secretagogue receptor (GHS-R) [13]. Circulating ghrelin is produced primarily in the stomach by X/A-like cells of the fundic glands, while the remainder originates in X/A-like cells of the small intestine [14]. Growth hormone secretagogue receptors are present in tissues other than the hypothalamus and pituitary, which indicates that ghrelin has other effects in addition to stimulating the release of growth hormone [15]. Indeed, besides the stimulation of GH release, ghrelin has also been described to have beneficial effect on gastrointestinal [16], cardiovascular [17], reproductive [18] and coagulation systems [19]. Recent studies have revealed that ghrelin may be an anti-inflammatory agent in many organs such as the rat ovary [20] and brain [21].

Although studies have shown that sulfite exposure leads to increased arachidonic acid levels in alveolar macrophages, changes in liver, heart and kidney PUFA levels following Na_2_S_2_O_5_ ingestion has not been investigated. This study was designed to determine changes in endogenous PUFA levels in rat peripheral organs and investigate whether ghrelin attenuates inflammatory pathways induced by Na_2_S_2_O_5_.

## Materials and methods

### Preparation of animals

All experimental protocols conducted on rats were performed in accordance with the standards established by the Institutional Animal Care and Use Committee at Akdeniz University Medical School. Male Wistar rats weighing 350–450 g were housed in stainless steel cages and were allowed free access to water and standard rat chow (Korkutelim Yem, Antalya, Turkey) containing 6.05% crude fat which included linoliec acid, linolenic acid, saturated fatty acids and monounsaturated fatty acids. Animals were maintained at 12 h light–dark cycles and a constant temperature of 23 ± 1°C at all times. Rats were randomly divided into four experimental groups which included control (n = 8); rats treated with sodium metabisulfite (Na_2_S_2_O_5_) (n = 10); rats treated with ghrelin (n = 10); rats treated with Na_2_S_2_O_5_ + ghrelin (n = 10). Control group received 1 ml/kg/day distilled water via gavage and 1 ml/kg/day saline via intraperitoneal injection as vehicle for 35 days. Sodium metabisulfite treated animals were given freshly prepared Na_2_S_2_O_5_ (100 mg/kg/day) solution via gastric gavage for 5 weeks as previously described [22]. We have shown that Na_2_S_2_O_5_ is efficiently absorbed at the given dose when administered via intragastric gavage and significantly increases plasma S-sulfonate levels [23]. Rat Ghrelin (GenScript, NJ, USA) was dissolved in distilled water (1 mg/ml) and stored at −20°C until the time of preparation for administration. Immediately before administration, ghrelin was diluted with 0.9% physiologic saline to a final concentration of 0.1 mg/ml. Ghrelin was given intraperitoneally (ip) at adose of 20 μg/kg for 35 days as previously described [24]. We have previously shown that the given dose and duration of ghrelin has protective effects against oxidative tissue injury and apoptosis in rats [24].

### Electrospray ionization mass spectrometry

Standards for arachidonic acid (AA, C20:4n-6), dihomo-gamma-linolenic acid (DGLA, C20:3n-6), eicosapentaenoic acid (EPA, C20:5n-3) and docosahexaenoic acid (DHA, C22:6n-3) were purchased from Sigma-Aldrich (St. Louis MO, USA). Deuterium labeled AA-d8 internal standard (5,6,8,9,11,12,14,15-AA-d8) was obtained from Santa Cruz Biotechnology (Santa Cruz, CA, USA). Solutions of AA, DGLA, EPA, DHA and AA-d8 standards were prepared in analytical grade methanol (Merck, Darmstadt, Germany). An optimized multiple reaction monitoring (MRM) method was developed using ultra-fast liquid chromatography (UFLC) coupled with tandem mass spectrometry (MS/MS). A UFLC system (LC-20 AD UFLC XR, Shimadzu Corporation, Japan) was coupled to a LCMS-8040 triple quadrupole mass spectrometer (Shimadzu Corporation, Japan). Chromatographic separations were carried out using Inertsil HPLC column (ODS-4, 2.1 × 100 mm, 3 μm; GL Sciences Inc. Tokyo, Japan) maintained at 40°C. DHA, EPA, AA and DGLA were separated using a gradient elution with a flow rate of 0.45 ml/min. Mobile phase solvent A was 10 mM ammonium acetate (Sigma-Aldrich, St. Louis, MO, USA) in water and solvent B was acetonitrile (Sigma-Aldrich, St. Louis, MO, USA). Gradient program was solvent B, 70% (0 min), 90% (3 min), 100% (3.01-4 min) and 70 % (4.01-8 min). MRM transitions and responses were automatically optimized for individual compounds in negative electrospray ionization (ESI). In the negative ESI-MS mode the precursor and product m/z values for AA, DHA, EPA, DGLA and AA-d8 are given in the results section. Responses to AA, DHA, EPA and DGLA were optimized to a linear calibration range from 100 ng/ml to 30 ug/ml and a sample analysis time of 8 minutes.

### Sample preparation for LC-MS/MS

Samples were prepared for LC-MS/MS analysis via a modified protocol as previously described [25,26]. All tissues were weighed and homogenized in ice-cold 50 mmol/L sodium phosphate buffer (pH 7.4). Homogenates were centrifuged (10,000 g for 15 min at 4°C) and supernatants were stored at −80°C. Briefly, in a glass test tube, 200 μl tissue supernatant was added to 200 μl AA-d8 internal standard solution. 1 ml of acetonitril/37% hydrochloric acid (Cayman, Ann Arbor, MI, USA) was added to the mixture in a 4:1 v/v. Tubes were capped with reusable teflon liner screw caps and samples were hydrolyzed by incubating at 90°C for 2 hours in a heating block (VLM, Bielefeld, Germany). After cooling down to room temperature, fatty acids were extracted with 2 ml of hexane. Samples were vortex-mixed for 20 seconds, left at room temperature for 5 minutes and centrifuged at 3000 rpm for 1 minute. The upper phase containing free fatty acids were transferred to glass tubes and evaporated at room temperature under a constant stream of nitrogen with height adjustable gas distribution unit (VLM, Bielefeld, Germany). Fatty acids were dissolved in 200 μl methanol–water (180:20, v/v) filtered via 0,2 μm polytetrafluoroethylene (PTFE) syringe filters (Whatman, GE Healthcare Bio-Sciences, Pittsburgh, USA) and transferred to autosampler vials (Vertical Chromatography, Nonthaburi, Thailand).

### Measurement of liver cyclooxygenase activity

Liver tissues were weighed and homogenized in 0.1 M ice-cold Tris–HCl buffer at pH 7.8 containing 1 mM EDTA. Tissue homogenates were centrifuged at 10,000 g for 15 minutes at 4°C and supernatants were kept at −80°C until assayed. Cyclooxygenase (COX) activity was measured using a COX activity assay kit (Cayman Chemical, Cat No: 760151 Ann Arbor, MI, USA) according to manufacturer’s instructions. The COX activity assay kit measures enzyme activity colorimetrically by monitoring the appearance of oxidized N,N,N*’*,N*’*-tetramethyl-p-phenylenediamine (TMPD) at 590 nm. One unit of enzyme activity was defined as the amount of enzyme that caused the oxidation of 1 nmol of TMPD per minute at 25°C.

### Measurement of liver Prostaglandin E_2_

Prostaglandin E_2_ (PGE2) was measured in tissue samples by a commercial enzyme immunoassay test kit (Cayman Chemical, Cat No: 514010 Ann Arbor, MI, USA) according to manufacturer’s instructions. Liver tissues were weighed and homogenized in 0.1 M ice-cold phosphate buffer at pH 7.4 containing 1 mM EDTA and 10 μM indomethacin. Tissue homogenates were centrifuged at 10,000 g for 15 minutes at 4°C and supernatants were kept at −80°C until assayed. Briefly, PGE2 present in the sample competes with acetylcholinesterase-labeled PGE2 antibody for binding sites on a goat polyclonal anti-mouse antibody. Following a wash to remove unbound materials, a substrate solution is added to the wells to determine the bound enzyme activity. The color development is stopped, and the absorbance is read at 412 nm. The intensity of the color is inversely proportional to the concentration of PGE2 in the sample. A standard curve of absorbance values of known PGE2 standards was plotted as a function of the logarithm of PGE2 standard concentrations (pg/ml) using the GraphPad Prism Software program for windows version 5,03. (GraphPad Software Inc). PGE2 concentrations in the samples were calculated from their corresponding absorbance values via the standard curve.

### Protein measurements

Protein concentrations were measured at 595 nm by a modified Bradford assay using Coomassie Plus reagent with bovine serum albumin as a standard (Pierce Chemical Company, Rockford, IL).

### Statistical analysis

Data were analyzed using Sigma Stat (version 2.03) statistical software for Windows, and a P value <0.05 was considered statistically significant. Statistical analysis for each measurement is described within the figure and table legends.

## Results

### Body weight increase

**Figure 1:**
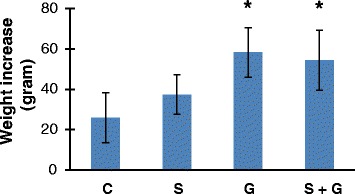
**Weight increase during the 5 week experimental period.** C, control; S, sodium metabisulfite; G, ghrelin; S + G, sodium metabisulfite + ghrelin. All values are mean ± SD. Statistical analysis was performed by one way analysis of variance and all pairwise multiple comparisons were via Tukey test. *, p < 0.05 vs. C and G.

We have recorded the body weight of all animals during the 5 week experimental period. We have seen a significant increase in the weight of animals treated with ghrelin. This data is shown in Figure 1. There was a significant increase in the weight of animals treated ghrelin. Weight increase (mean ± SD) measured in experimental groups were as follows: Control (n = 8), 26,00 ± 12,27; Na_2_S_2_O_5_ (n = 10), 37,50 ± 9,79; Ghrelin (n = 10), 58,33 ± 12,25 and Na_2_S_2_O_5_ + Ghrelin (n = 10), 54,44 ± 14,88 grams.

### ESI-MS spectra

**Figure 2:**
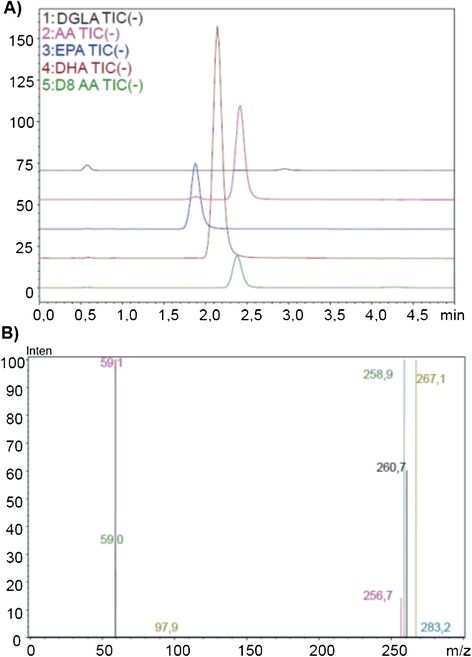
**ESI-MS spectra. A)** Representative negative ion mode spectra of DGLA, Dihomo-gamma-linolenic acid; AA, Arachidonic acid; EPA, Eicosapentaenoic acid; DHA, Docosahexaenoic acid. **B)** Tandem mass spectra.

The precursor and product m/z values for analyzed PUFAs were as follows: DGLA (C20: 3n6), precursor m/z: 304.80, product m/z: 59.00, 260.70; AA (C20: 4n6), precursor m/z: 303.10, product m/z: 59.00, 258.90; EPA (C20:5n3), precursor m/z: 301.10, product m/z: 59.10, 256.70; DHA (C22: 6n3): precursor m/z: 327.10, product m/z: 59.10, 283.20; AA-d8, precursor m/z: 311.10, product m/z: 59.10, 97.90, 267.10. Figure 2A shows representative negative ion mode spectra obtained from measured PUFAs. As shown in Figure 2A, retention time of time of EPA (C20: 5n3), DHA (C22: 6n3), AA (C20: 4n6), AA-d8 and DGLA (C20: 3n6) was 1.869, 2.131, 2.391, 2.329 and 2.911 minutes, respectively. Figure 2B shows tandem mass spectra obtained by collision-induced dissociation of precursor ions. The m/z values of product ions correspond to endogenous C20: 5n3, C20: 4n6, C20: 3n6 and C22: 6n3. The deuterium-labeled internal standard fatty acid peak is indicated at m/z values 267.1.

### Levels of polyunsaturated fatty acids

**Figure 3:**
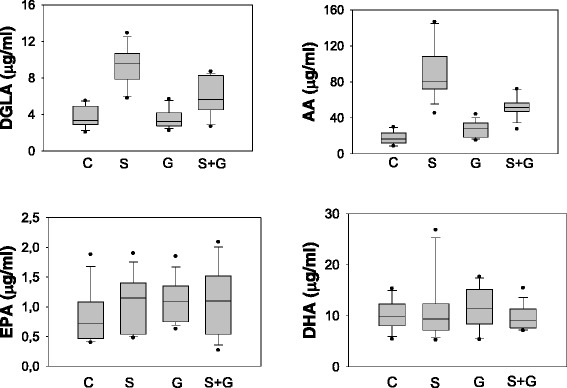
**Distribution of polyunsaturated fatty acids in the liver of experimental groups.** C, control; S, sodium metabisulfite; G, ghrelin; S + G, sodium metabisulfite + ghrelin. DGLA, Dihomo-gamma-linolenic acid; AA, Arachidonic acid; EPA, Eicosapentaenoic acid; DHA, Docosahexaenoic acid.

Levels of endogenous PUFAs measured in liver, heart and kidney tissues are given in Tables 1, 2 and 3, respectively. DGLA and AA levels were increased in liver tissue following sodium metabisulfite treatment compared to control and ghrelin treated groups. No significant change was observed in liver EPA and DHA amount following sodium metabisulfite treatment. Ghrelin treatment significantly decreased DGLA and AA levels in sodium metabisulfite treated rats. AA/DHA and AA/EPA ratio were significantly increased in liver tissue following sodium metabisulfite treatment compared to control, ghrelin and Na_2_S_2_O_5_ + ghrelin groups (Table 1). No significant change was observed in heart and kidney PUFA levels, AA/DHA and AA/EPA ratio (tables 2 and 3, respectively). Box plot graph data of AA (C20:4n-6), DGLA (C20:3n-6), EPA (C20:5n-3) and DHA (C22:6n-3) distribution in liver of all experimental groups are shown in Figure 3. The boundary of the box closest to zero indicates the 25th percentile, the line within the box marks the median, and the boundary of the box farthest from zero indicates the 75th percentile. Whiskers above and below the box indicate the 90th and 10th percentiles.

**Table 1:** **Analysis of polyunsaturated fatty acids in liver tissue**

**Parameter**	**Control (** ***n = 8*** **)**	**Na**_**2**_ **S**_**2**_ **O**_**5**_ **(** ***n = 10*** **)**	**Ghrelin (** ***n = 10*** **)**	**Na**_**2**_ **S**_**2**_ **O**_**5**_ **+ Ghrelin (** ***n = 10*** **)**
**DGLA (C20:3n6)**	3.73 ± 1.26	9.44 ± 2.32^a^	3.65 ± 1.15	5.98 ± 2.52^b^
**AA (C20:4n6)**	17.73 ± 7.74	90.44 ± 32.63^c,d^	29.43 ± 9.09	54.89 ± 10.57^b,e^
**EPA (C20:5n3)**	0.85 ± 0.50	1.11 ± 0.47	1.12 ± 0.38	1.13 ± 0.60
**DHA (C22:6n3)**	10.14 ± 3.29	11.96 ± 7.45	11.42 ± 4.46	9.74 ± 2.56
**AA/DHA**	3.35 ± 1.17	9.33 ± 3.02^f^	3.05 ± 1.02	4.01 ± 0.71
**AA/EPA**	27.66 ± 14.21	87.66 ± 37.24^f^	26.60 ± 7.67	34.36 ± 7.64

Values are expressed as mg fatty acid/g tissue protein. Data are reported as mean ± SD. Na_2_S_2_O_5_, sodium metabisulfite; DGLA, Dihomo-gamma-linolenic acid; AA, Arachidonic acid; EPA, Eicosapentaenoic acid; DHA, Docosahexaenoic acid. Statistical analysis was performed by one way analysis of variance with all pairwise multiple comparisons via Tukey test or Kruskal-Wallis One Way Analysis of Variance on Ranks with all pairwise multiple comparison procedures by Dunn's Method.

^a^p ≤ 0.001 Na_2_S_2_O_5_ vs. control, ghrelin, Na_2_S_2_O_5_ + ghrelin.

^b^p < 0.05 Na_2_S_2_O_5_ + ghrelin vs. Ghrelin.

^c^p < 0.001 Na_2_S_2_O_5_ vs. control, ghrelin.

^d^p = 0.002 Na_2_S_2_O_5_ vs. Na_2_S_2_O_5_ + ghrelin.

^e^p = 0.002 Na_2_S_2_O_5_ + ghrelin vs. control.

^f^p < 0.05 Na_2_S_2_O_5_ vs. control, ghrelin, Na_2_S_2_O_5_ + ghrelin.

**Table 2:** **Analysis of polyunsaturated fatty acids in heart tissue**

**Parameter**	**Control (** ***n = 8*** **)**	**Na**_**2**_ **S**_**2**_ **O**_**5**_ **(** ***n = 10*** **)**	**Ghrelin (** ***n = 10*** **)**	**Na**_**2**_ **S**_**2**_ **O**_**5**_ **+ Ghrelin (** ***n = 10*** **)**
**DGLA (C20:3n6)**	0.15 ± 0.06	0.18 ± 0.06	0.16 ± 0.08	0.14 ± 0.07
**AA (C20:4n6)**	2.56 ± 1.02	2.95 ± 0.56	2.46 ± 1.10	2.28 ± 1.01
**EPA (C20:5n3)**	0.07 ± 0.02	0.06 ± 0.03	0.05 ± 0.04	0.05 ± 0.02
**DHA (C22:6n3)**	2.27 ± 0.98	2.65 ± 0.92	2.81 ± 1.46	1.81 ± 0.98
**AA/DHA**	1.16 ± 0.16	1.09 ± 0.18	0.90 ± 0.13	1.24 ± 0.30
**AA/EPA**	49.52 ± 12.76	44.50 ± 14.70	47.57 ± 10.43	54.11 ± 17.80

Values are expressed as mg fatty acid/g tissue protein. Data are reported as mean ± SD. Na_2_S_2_O_5_, sodium metabisulfite; DGLA, Dihomo-gamma-linolenic acid; AA, Arachidonic acid; EPA, Eicosapentaenoic acid; DHA, Docosahexaenoic acid. Statistical analysis was performed by one way analysis of variance or Kruskal-Wallis One Way Analysis of Variance on Ranks.

**Table 3:** **Analysis of polyunsaturated fatty acids in kidney tissue**

**Parameter**	**Control (** ***n = 8*** **)**	**Na**_**2**_ **S**_**2**_ **O**_**5**_ **(** ***n = 10*** **)**	**Ghrelin (** ***n = 10*** **)**	**Na**_**2**_ **S**_**2**_ **O**_**5**_ **+ Ghrelin (** ***n = 10*** **)**
**DGLA (C20:3n6)**	1.00 ± 0.47	1.18 ± 0.60	0.76 ± 0.17	0.77 ± 0.57
**AA (C20:4n6)**	10.70 ± 5.39	12.69 ± 5.51	7.11 ± 1.94	9.18 ± 6.32
**EPA (C20:5n3)**	0.45 ± 0.19	0.48 ± 0.13	0.36 ± 0.11	0.43 ± 0.19
**DHA (C22:6n3)**	1.71 ± 0.97	2.13 ± 0.91	1.60 ± 1.27	1.30 ± 0.34
**AA/DHA**	6.91 ± 2.76	6.04 ± 1.44	6.13 ± 1.19	5.60 ± 0.41
**AA/EPA**	24.03 ± 7.01	27.84 ± 13.80	20.69 ± 4.27	20.50 ± 3.95

Values are expressed as mg fatty acid/g tissue protein. Data are reported as mean ± SD. Na_2_S_2_O_5_, sodium metabisulfite; DGLA, Dihomo-gamma-linolenic acid; AA, Arachidonic acid; EPA, Eicosapentaenoic acid; DHA, Docosahexaenoic acid. Statistical analysis was performed by one way analysis of variance or Kruskal-Wallis One Way Analysis of Variance on Ranks.

### Liver cyclooxygenase activity

**Figure 4:**
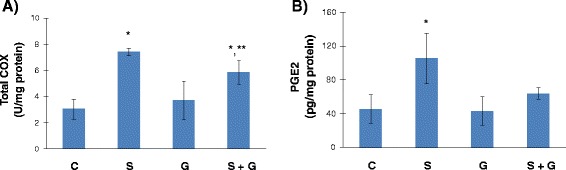
**Liver cyclooxygenase and prostaglandin E2 levels.** **A)** Liver cyclooxygenase activity. C, control; S, sodium metabisulfite; G, ghrelin; S + G, sodium metabisulfite + ghrelin. All values are mean ± SD. Statistical analysis was performed by one way analysis of variance and all pairwise multiple comparisons were via Tukey test. *, p < 0.001 vs. C and G. **, p = 0.005 vs. S. **B)** Liver prostaglandin E_2_ levels. All values are mean ± SD. Statistical analysis was performed by one way analysis of variance and all pairwise multiple comparisons were via Tukey test. *, p < 0.001 vs. C, G and S + G.

Total COX activity, as shown in Figure 4A, was significantly increased in liver tissue following sodium metabisulfite treatment when compared to control and ghrelin treated groups. Ghrelin treatment significantly decreased total COX activity in Na_2_S_2_O_5_ treated rats. Total COX activity (mean ± SD) measured in experimental groups were as follows: Control, 3.06 ± 0.77; Na_2_S_2_O_5_, 7.43 ± 0. 29; Ghrelin, 3.72 ± 1.47 and Na_2_S_2_O_5_ + Ghrelin, 5.87 ± 0,90 U/mg protein.

### Liver prostaglandin E_2_ levels

Liver PGE2 levels, as shown in Figure 4B, was significantly increased in liver tissue following sodium metabisulfite treatment when compared to control, ghrelin, and Na_2_S_2_O_5_ + ghrelin treated groups. PGE2 levels (mean ± SD) measured in experimental groups were as follows: Control, 45,64 ± 16,91; Na_2_S_2_O_5_, 105,56 ± 29,90; Ghrelin, 43,40 ± 16,68 and Na_2_S_2_O_5_ + Ghrelin, 64,09 ± 6,96 pg/mg protein.

## Discussion

To the best of our knowledge, this study is the first to measure endogenous DGLA (C20:3n-6), AA (C20:4n-6), DHA (C22:6n-3) and EPA (C20:5n-3) levels in rat peripheral organs following Na_2_S_2_O_5_ ingestion. This study makes the novel observation that ingestion of 100 mg/kg/day Na_2_S_2_O_5_ for 5 weeks results in significantly increased n-6 PUFA levels, AA/DHA and AA/EPA ratio in the liver and leads to significantly increased tissue activity of COX and PGE2 levels. It also shows that ghrelin decreases n-6 PUFA levels and reduces COX and PGE2 levels in liver tissue of sodium metabisulfite treated rats.

The dose of sulfite administered in this study was determined with reference to our previous studies designed to investigate the effects of high level of sulfite intake [22-24]. The amount of ingested sulfite is usually expressed as SO_2_ equivalents (SDE) and with reference to the reported theoretical yield of 67.39 % SO_2_ from Na_2_S_2_O_5_ [27,28], the given dose of 100 mg Na_2_S_2_O_5_/kg/day was equivalent to 67.39 mg/kg/day SO_2_. The Joint Expert Committee in the World Health Organization (WHO) has established an acceptable daily intake (ADI) level for sulfites which is reported to be 0.7 mg/kg body weight, expressed as SO_2_ [28]. Considering that many foods such as sausage, dried fruit, beer and wine contain sulfite, the ADI level can easily be exceeded. It is possible to consume 180–200 mg/day sulfite from foods and beverages in a single day or meal [2,29].

Hepatic oxidation of exogenous sulfite is diffusion limited [6]. That is to say the liver metabolizes a constant fraction of sulfite it receives, but a limited amount will pass through the organ and enter the systemic circulation [2]. Mammalians contain sulfite oxidase (SOX), which catalyzes the oxidative detoxification of sulfite in order to protect the cells from sulfite toxicity. It was shown that rats have a 10–20-fold greater SOX activity than humans [5]. Ingested sulfites also undergo a nonenzymatic reaction with disulfide bonds generating glutathione S-sulfonate [4] and thus, plasma S-sulfonate level is commonly used as a biomarker of ingested sulfite [30]. The rhesus monkey and the rabbit accumulate plasma S-sulfonates much more readily than rat, while the mouse produce little under the same test conditions [31]. Unlike rodents, plasma S-sulfonate levels can be readily detected under basal condition in humans [32]. It was previously shown by our group that Na_2_S_2_O_5_ is efficiently absorbed at the given dose (100 mg/kg/day) when administered via intragastric gavage and significantly increases plasma S-sulfonate levels in rats [23,33].

The observed increase in liver n-6 PUFA levels, AA/DHA and AA/EPA ratio support previous studies which have shown that long-term in vivo exposure to sulfite aerosols induces inflammatory reactions [34] and that alveolar macrophages incubated with sulfite generate significantly increased amounts of arachidonic acid (AA) and AA-derived eicosanoids [10]. Mammalians can produce many fatty acids except the two essential PUFAs which include linoleic acid (LA, C18:2n6) and alpha-linolenic acid (ALA, C18:3n3). Linoleic acid is the precursor of n-6 series of PUFAs including DGLA (C20:3n-6) and AA (C20:4n-6) while ALA is the precursor of n-3 series of PUFAs which include DHA (C22:6n-3) and EPA (C20:5n-3). Competition between n-6 and n-3 fatty acids occurs in the production of eicosanoids by stereospecific lipid-oxidizing enzymes cylooxygenase (COX) and lipoxygenase (LOX) [35]. Eicosanoids, derived mainly from AA (C20:4n-6) are key mediators and regulators of inflammation. They include prostaglandins (PGs), thromboxanes (TXs) and leukotrienes (LTs) [36]. Eicosanoids derived from n-3 PUFAs such as EPA, C20:5n-3) have anti-inflammatory properties, attributed to their ability to inhibit the formation of n-6 PUFA-derived eicosanoids [36]. Resolvins and protectins generated from EPA (C20:5n-3) and DHA (C22:6n-3) display potent antiinflammatory properties and are recognized in the resolution of inflammation [37]. Hence, increased AA to EPA or AA to DHA ratio indicates more precursor for the synthesis of highly inflammatory eicosanoids.

We have seen that the given dose of sodium metabisulfite treatment caused no significant change in PUFA levels in heart and kidney tissues of rats while it increased DGLA (C20: 3n-6) and AA (C20: 4n-6) levels in the liver. This discrepancy is likely to result from the metabolism of ingested sulfite. As stated previously, exogenous sulfites are presented to the liver’s biotransformation system for processing and elimination and their oxidation is diffusion limited [6]. However, when in excess amount they can stress the detoxification capability of the liver or be partially processed and accumulate in the liver [2]. This can lead to increased liver stores of these toxic compounds and cause tissue injury.

Activity of COX, the initial enzyme of prostaglandin synthesis was also measured in liver tissue following sodium metabisulfite treatment. Cyclooxygenase is the rate-limiting enzyme in the production of prostanoids from arachidonic acid. Research has showed that the COX/prostanoid pathway is activated in hepatic diseases and liver stress reaction, such as alcoholic liver disease [38], liver fibrogenesis [39], viral hepatitis C [40] and liver ischemic/reperfusion injury [41] causing liver damage manifested as inflammation, necrosis and fatty liver. In agreement with increased liver COX activity, we have also measured significantly increased liver PGE2 levels following sodium metabisulfite ingestion. Arachidonic acid is a precursor of PGE2 synthesis and PGE2. It is produced during inflammatory responses and mediates a variety of both innate and adaptive immune responses through different receptor subtypes [42]. Acetylsalicylic acid (ASA) is a COX inhibitor and different from other cyclooxygenase inhibitors it enhances the formation of anti-inflammatory and pro-resolution lipoxins derived from arachidonic acid as well as resolvins from n-3 PUFAs such as docosahexaenoic acid (DHA). In a recent study, the effect of ASA was examined on murine dextran sodium sulfate-induced colitis. The results showed that ASA reduced the severity of dextran sodium sulfate-induced colitis and increased the formation of anti-Inflammatory lipid mediators [43].

We have seen that ghrelin decreased both n-6 PUFA levels and attenuated n-6 inflammatory pathway in liver tissue following high level of sulfite intake. These observations are in agreement with studies reporting the beneficial effect of ghrelin on the gastrointestinal system [16] and support the role of ghrelin as an anti-inflammatory agent in many organs such as the rat ovary [20] and brain [21].

Ghrelin has been proposed as an antioxidant agent recently in several studies [18,21]. Obay at al. demonstrated that ghrelin prevents lipid peroxidation and decrease of antioxidant enzyme activities against pentylenetetrazole induced oxidative stress in erythrocytes, liver and brain in rats [21]. Moreover, several studies showed that, ghrelin significantly increases the activity of antioxidant enzymes such as GPx, SOD and CAT and decreases the concentration of malondialdehyde, a product of lipid peroxidation [18]. The antioxidant property of ghrelin was also demonstrated by attenuation of oxidative stress in gastric mucosa triggered by Na_2_S_2_O_5_ [24].

Ghrelin is a neuropeptide that can be produced by immune cells especially under inflammatory conditions [44]. Anti-inflammatory neuropeptides emerge as a new strategy to manage inflammation-based diseases. In mammals, several lines of evidence indicate that ghrelin stimulates corticosteroid release in rats [45]. Ghrelin can also signal through the activation of cAMP/protein kinase A (PKA) pathway, which is considered as an immunosuppressive signal [46]. Through the elevation of intracellular cAMP, it can down-regulate the activation of several transduction pathways and transcription factors essential for the transcriptional activation of most of the inflammatory cytokines [44]. Observed findings herein support the anti-inflammatory actions of ghrelin reported previously.

## Conclusion

In summary, we report that liver AA (C20:4n-6), DGLA (C20:3n-6), AA/DHA and AA/EPA ratio are significantly increased in rats following high level of sulfite intake and that sulfite ingestion leads to significantly increased liver activity of COX and PGE2 levels. We also show that ghrelin decreases n-6 PUFA levels and reduces COX and PGE2 levels in liver tissue of sodium metabisulfite treated rats. Further studies are needed to clarify mechanisms by which ghrelin regulates the n-6 inflammatory pathway in liver tissue.

## Abbreviations

AA: Arachidonic acid
ADI: Acceptable daily intake
ALA: Alpha-linolenic acid
COX: Cyclooxygenase
DGLA: Dihomo-gamma-linolenic acid
DHA: Docosahexaenoic acid
EPA: Eicosapentaenoic acid
ESI: Electrospray ionization
GH: Growth hormone
GHS-R: GH secretagogue receptor
HSO_3_^−^: Bisulfite
LA: Linoleic acid
LTB4: Leukotriene B4
LTs: Leukotrienes
MRM: Multiple reaction monitoring
MS/MS: Tandem mass spectrometry
n-3: Omega-3
n-6: Omega-6
Na_2_S_2_O_5_: Sodium metabisulfite
PAF: Platelet-activating factor
PGE_2_: Prostaglandin E_2_
PGs: Prostaglandins
PTFE: Polytetrafluoroethylene
PUFAs: Polyunsaturated fatty acids
SDE: SO_2_ equivalents
SO_2_: Sulfur dioxide
SO_3_^2−^: Sulfite
SOX: Sulfite oxidase
TMPD: N,N,N*’*,N*’*-tetramethyl-p-phenylenediamine
TXB_2_: Thromboxane B_2_
TXs: Thromboxanes
UFLC: Ultra fast-liquid chromatography
WHO: World Health Organization
